# A novel risk score for venous thromboembolism in lung cancer patients: a retrospective cohort study

**DOI:** 10.12688/f1000research.138878.1

**Published:** 2023-10-20

**Authors:** Houda Rouis, Chirine Moussa, Islem mejri, Soumaya Debbiche, Nourchene Khalfallah, Lenda Ben Hmida, Amel Khattab, Zied Moetamri, Mohamed Lamine Megdiche, Hela Kamoun, Sonia Maâlej

**Affiliations:** 1 Pneumology 1, Abderrahmen Mami Pneumology and Phthisiology Hospital, Ariana, Ariana, Tunisia; 2 pneumology, Military Hospital of Tunis, Tunis, 1008, Tunisia; 3 Ibn Nafis, Abderrahmen Mami Pneumology and Phthisiology Hospital, Ariana, Ariana, Tunisia

**Keywords:** Lung cancer, Venous thromboembolism, risk score

## Abstract

**Background:** Venous thromboembolism (VTE) is a common and potentially fatal complication in patients with lung cancer. This study aimed to develop and validate a risk score for early prediction of VTE in these patients.

**Methods:** Four hundred and one patients with lung cancer from three pulmonology departments hospitalized between January 2011 and December 2021 were retrospectively assessed. The population was divided into two groups: a Development Group (182 patients) and a validation group (199 patients). In the development group, the risk score system was developed, via univariate and multivariate analyses, based on demographic and clinicopathological variables; it was then validated in the validation group.

**Results:** The incidence of VTE was 26.8% in the development group. It was 25.8%, and 27.6% in the internal and external validation groups, respectively. Hemoglobin level <10g/l, metastasis, histological type poorly or undifferentiated non-small cell carcinoma, and active smoking were the items of the risk score system. This score allowed proper stratification of patients with either high or low risk of VTE in the development group (c statistic =0.703). The patients in the development group were classified into 3 risk groups: low risk (scores 0-1), moderate risk (scores 2-3), and high risk (scores 4-5). When validated in the validation group, there was a moderate loss of predictive power of the score (c statistic=0.641), but the categorization of the patients by the score remained clinically useful.

**Conclusions:** This risk score requires prospective validation studies on a nationwide scale in order to use it as a valid tool for the prevention of VTE in lung cancer.

## Introduction

Patients diagnosed with cancer are at a higher risk of developing venous thromboembolism (VTE) compared to the general population, with reported incidence rates ranging from 20% to 30%. ^1^ Among cancer types, lung cancer is particularly associated with thrombosis, with incidence rates ranging from 14% to 30. ^2^

The risk of thrombosis is significantly increased in cancer patients, by 4 to 12 times higher compared to individuals without cancer, and further elevated to 6.5 to 23 times with the addition of chemotherapy or targeted therapy. ^3^^–^^5^

Various factors contribute to this increased risk, including patient-related factors such as advanced age, previous history of VTE, and obesity; tumor-related factors such as cancer type, stage, and aggressivenessr; and treatment-related factors like surgery, radiation, or systemic anticancer. ^1^^,^^6^

The reported incidence of VTE in the literature varies, possibly due to differences in study design, selection of study participants, varying definitions of VTE, and the exclusion of patients with previous thrombosis from most clinical trials. ^1^

The correlation between cancer and venous thromboembolism (VTE) has been extensively established and is known to have deleterious effects on both morbidity and mortality among cancer patients. ^5^

VTE, including pulmonary embolism (PE) and deep vein thrombosis (DVT), as well as arterial thromboembolism, rank as the second most common cause of death in cancer patients. ^1^ Moreover, cancer patients with superficial vein thrombosis (SVT) face a high risk of death comparable to those with DVT. ^6^

Preventing VTE is an essential component of managing lung cancer patients to improve their prognosis. Therefore, several medical scoring systems have been developed to predict the risk of VTE, such as the Khorana, Caprini, Vienna CATS, PROTECHT, and COMPASS-CAT scores. ^7^ Nevertheless, these risk prediction scores have been developed with inherent limitations and have undergone limited external validation in lung cancer patients. Additionally, their applicability to the Tunisian population is restricted. Consequently, it is a priority to develop and validate a score specifically tailored to the Tunisian population. This will facilitate the guidance of prophylactic anticoagulation in patients at risk of VTE.

## Methods

### Sample size calculation

Considering the objective of reducing the risk of VTE by 10% through the implementation of a VTE predictive score to guide prophylactic anticoagulation in lung cancer patients, it was determined that a sample size of 360 patients would be necessary to attain statistical significance (power: 0.8; alpha: 0.05), as calculated using a predictive formula. ^8^

### Source of data

We conducted a multicenter retrospective cohort study during the period between January 2011 and December 2021, involving the records of 410 patients who were diagnosed with lung cancer of all stages and who were hospitalized in the Department 1 or Ibn Néfis Department of Abderrahmane Mami Hospital of Ariana, or in the Pulmonary Department of the Principal Military Hospital of Tunis.

### Participants

**Ethics approval**

The study was approved by the ethics committee of abderrahmane mami hospital Under the approval number 27/2023. Written informed consent was collected from the patients. Anonymity was respected during data treatment.

*Inclusion criteria*

We enrolled all hospitalized patients whose diagnosis of primary lung cancer was histologically confirmed and who required hospitalization.

*Non-inclusion criteria*

We did not include the patients whose diagnosis of cancer has not been histologically confirmed or whose diagnosis of secondary lung cancer has been retained.

*Exclusion criteria*

Patients with prior history of other malignant tumors, acute myocardial infarction, and acute stroke were excluded from the study. Patients with inoperable records (many missing data) were also excluded.

### Data collection

The data collected included: demographic and clinicopathological data (age, gender, BMI, WHO status, smoking, hypertension, diabetes, dyslipidemia, coronary artery disease, heart failure, and surgical history), biological data (blood cell count (White blood cells, Hemoglobin, Platelets), C-reactive Protein (CRP), and creatinine), lung cancer data (histological type, TNM classification, length of follow-up, surgical treatment, chemotherapy, regimen of chemotherapy (platinum-based chemotherapy), and radiation), and VTE data (occurrence of PEor DVT, and the time to their onset).

### Outcomes

The event is defined as the occurrence of a VTE, which includes DVT, SVT, and/or PE.

SVT is a thrombosis occurring in a superficial vein. It is usually caused by an inflammatory reaction in the wall of the thrombosed vein. The diagnosis is strongly suggested clinically and confirmed by Doppler ultrasound.

DVT is characterized by the presence of a blood clot that completely or partially obstructs the blood flow in the deep venous system, most frequently in the lower limbs. The diagnosis is based on venous Doppler ultrasound.

A PE occurs when a thrombus (blood clot) obstructs an artery in the lung, resulting in impaired blood flow. The diagnosis of PE is confirmed through a thoracic angio-scan.

### Statistical analysis

The patients were randomly allocated to two groups: the development group (182 patients) and the validation group (199 patients). The development group was used to establish the scoring system, while the validation group was used to validate the developed score.

Random numbers were generated based on the sequence of medical record numbers, and subsequent grouping was determined by ranking the random numbers using SPSS software. Data entry and analysis were conducted using IBM SPSS 23.0 software. Categorical variables were analyzed by calculating frequencies and percentages, and Pearson’s Chi-squared test was employed for frequency comparisons.

The score development process comprised two steps using the development group’s database. Firstly, a univariate analysis was conducted to identify the risk factors for preoperative VTE. Secondly, variables with a significance level of p<0.05 from the first step were entered into a multivariate analysis using multiple binary logistic regressions. Odds ratios (ORs) and 95% confidence intervals were calculated. Independent variables were included in the regression model if their significance level was less than 0.25. Variables with a significance level of p<0.05 and a 95% confidence interval were considered predictive factors for VTE risk and constituted the components of the score. The weighting of these factors was determined by dividing the β coefficients by the absolute value of the smallest regression coefficient, rounding the result to the nearest integer. The sum of the weighted factors constituted the patient’s total risk score, which was calculated for both the development and validation groups to classify patients into risk groups.

The cut-offs were fixed using the ROC curve analysis (receiver operating characteristic curve). Once a cut-off has been specified, the calculation of the predicted incidence allows a better classification of the patients into risk groups (low, moderate, and high).

When our risk score was developed, its validation was done over two steps. The first step consists of the evaluation of the internal validity, which was done by studying the discrimination using the C statistic via the ROC curve analysis and the calibration via the Hosmer-Lemeshow test. The second step was based on the evaluation of the external validity. A total of 1000 bootstrap samples were selected from the database of the validation group to recalculate the discrimination and calibration of the risk model. For all statistical tests, the two-sided significance level was set at 0.5.

## Results

### Population characteristics and incidence of VTE

**Figure 1.**
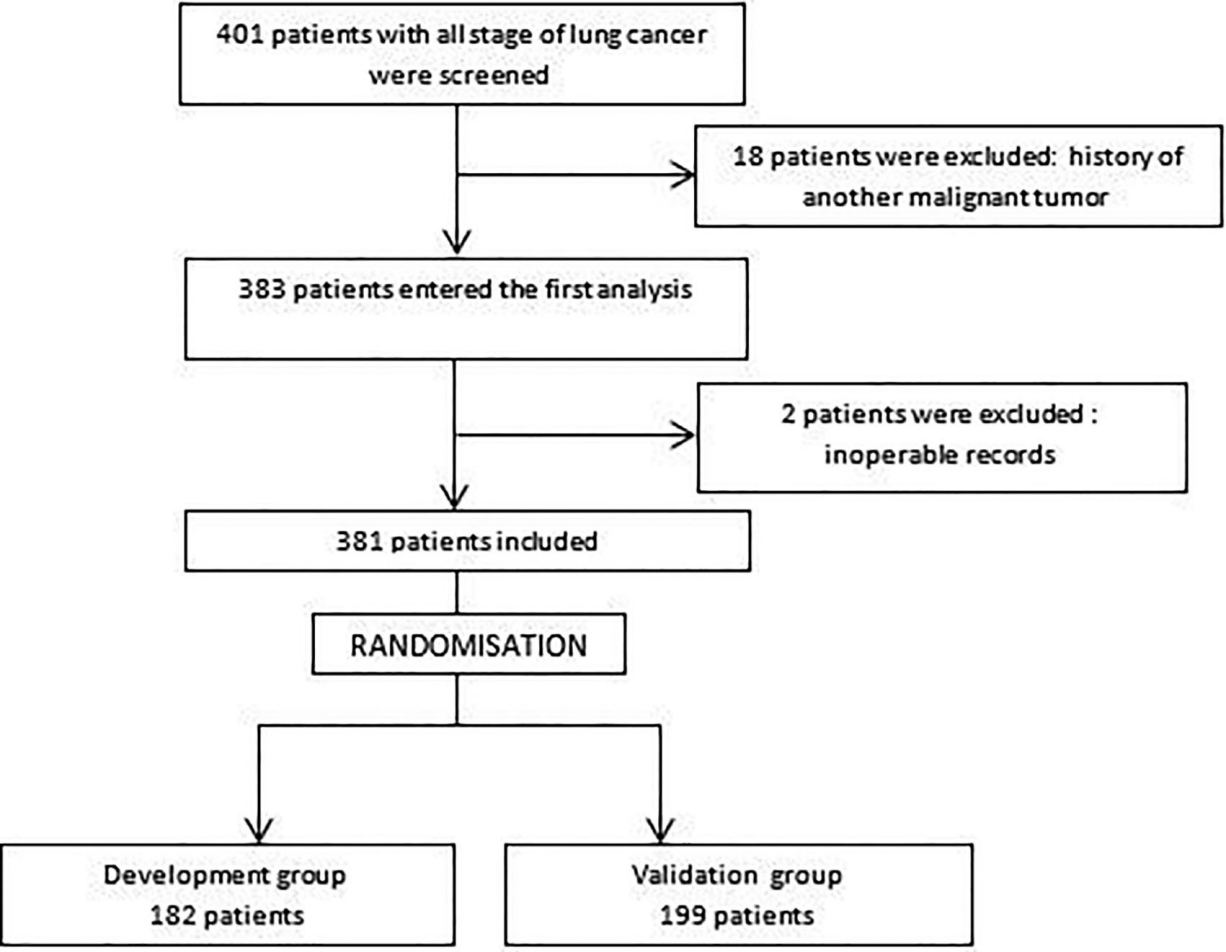
Flow chart of patient inclusion and exclusion.

A total of 381 patients were included in our study after the exclusion of 20 patients ( Figure 1). Thirty-nine patients had lung resection. Three hundred and nine patients (81.8% of cases) received first-line chemotherapy, which was combined with curative radiotherapy in 32.4% of cases.

**Figure 2.**
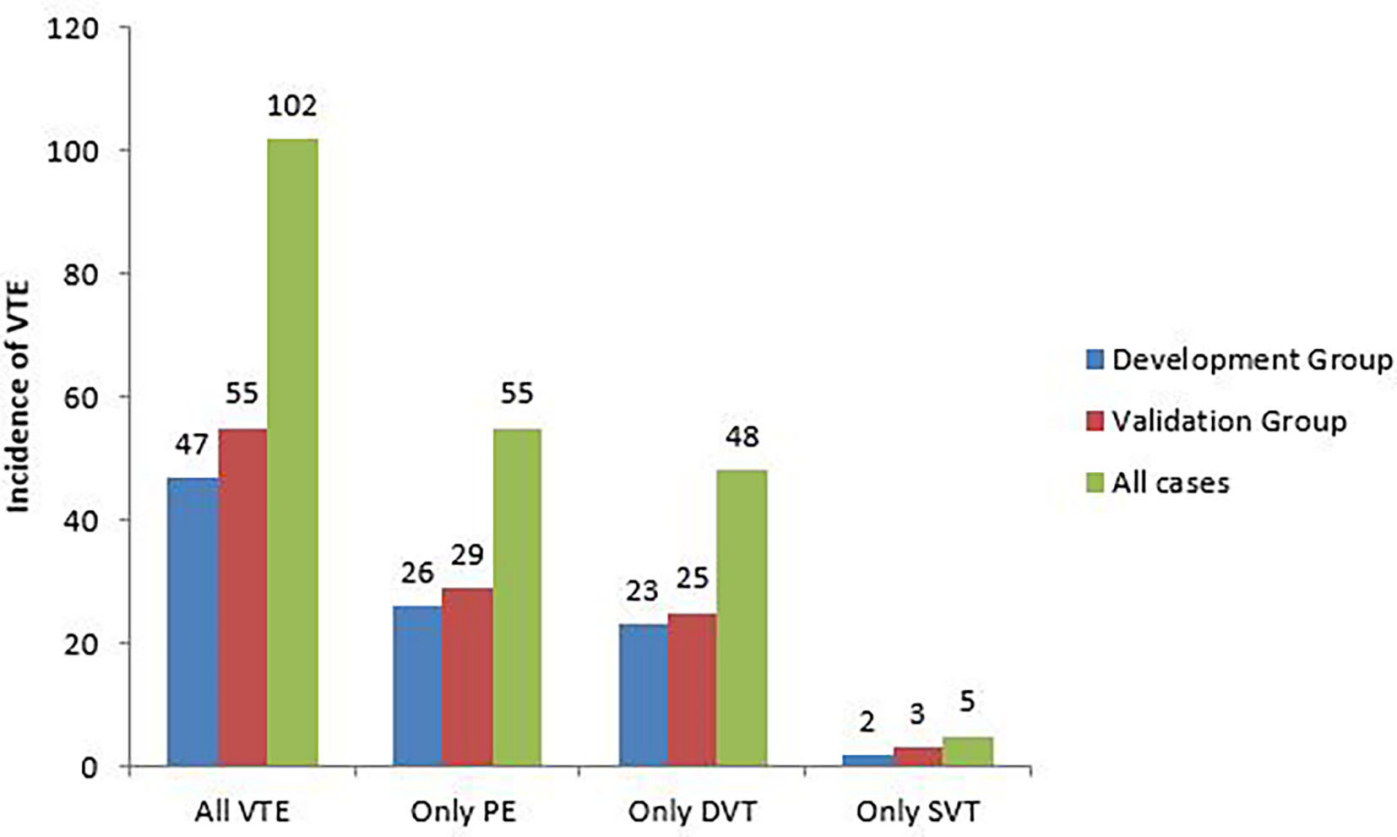
Incidence of VTE in patients followed for lung cancer.

The incidence of VTE was 26.8% (102/381). It was similar in the development and validation groups (25.8% vs 27.6%; p=0.690). There was no significant difference in the incidence of pulmonary embolism (PE) (14.3% vs 14.6%; p=0.954), DVT (12.6% vs 12.5%; p=0.966), or SVT (1.1% vs 1.5%; p=0.731) between the 2 groups ( Figure 2).

The main characteristics of the two databases are summarized in Table 1.

**Table 1.** Characteristics of patients in the development group and the validation group.

Variables	Development group n=182	Validation group n=199	χ ^2^	p
**Sex**				
Female	30 (16.5)	21 (10.6)	2.884	0.089
Male	152 (83.5)	178 (89.4)		
**Age (≥65 years)**				
Yes	92 (50.5)	76 (38.2)	5.890	0.015
No	90 (49.5)	123 (61.8)		
**BMI (≥25 kg/m** ^**2**^ **)**				
Yes	32 (17.6)	51 (25.6)	2.561	0.110
No	106 (58.2)	111 (55.8)		
**WHO status (≥2)**				
Yes	29 (15.9)	28 (14.1)	0.283	0.595
No	152 (83.5)	171 (85.9)		
**TNM stage**				
I-II	17 (9.3)	15 (7.5)	0.402	0.526
III-IV	165 (90.7)	184 (92.5)		
**Lymph node status (≥N2)**				
Yes	131 (72)	127 (69.8)	0.092	0.761
No	51 (28)	50 (37.9)		
**Metastasis**				
Yes	113 (62.1)	130 (65.3)	0.432	0.511
No	69 (37.9)	69 (34.7)		
**Histological type**				
Small-cell carcinoma	24 (13.2)	27 (13.6)	0.012	0.913
Poorly or undifferentiated	21 (11.5)	19 (9.5)	0.401	0.527
non-Small-cell carcinoma				
Squamous cell Carcinoma	48 (26.4)	46 (23.1)	0.543	0.461
Adenocarcinoma	84 (46.2)	95 (47.7)	0.096	0.757
Large cell carcinoma	3 (1.6)	7 (3.5)	1.300	0.254
**Active smoking**				
Yes	101 (55.5)	115 (57.8)	0.204	0.652
No	81 (45.5)	84 (42.2)		
**Hypertension**				
Yes	35 (19.2)	24 (12.1)	3.735	0.053
No	147 (80.8)	175 (87.9)		
**Diabetes mellitus**				
Yes	26 (14.3)	33 (16.6)	0.383	0.536
No	156 (85.7)	166 (83.4)		
**Dyslipidemia**				
Yes	9 (4.9)	9 (4.5)	0.038	0.846
No	173 (95.1)	190 (95.5)		
**Coronaropathy**				
Yes	17 (9.3)	14 (7)	0.676	0.411
No	165 (90.7)	185 (93)		
**Cardiac failure**				
Yes	6 (3.3)	5 (2.5)	0.208	0.648
No	176 (96.7)	195 (97.5)		
**Renal failure**				
Yes	3 (1.6)	1 (0.5)	1.201	0.273
No	179 (98.4)	198 (99.5)		
**History of surgery**				
Yes	43 (23.6)	52 (26.1)	0.352	0.553
No	139 (76.4)	146 (73.4)		
**WBC (≥10 × 10** ^**9**^ **/l)**				
Yes	70 (38.5)	102 (51.3)	6.781	0.009
No	112 (61.5)	97 (48.7)		
**Hb (<10 g/l)**				
Yes	22 (12.1)	20 (10.1)	0.402	0.526
No	160 (87.9)	179 (89.9)		
**Platelets (≥300 × 10** ^**9**^ **/l)**			0.034	0.854
Yes	95 (47.8)	100 (50.3)		
No	87 (52.2)	99 (49.7)		
**Creatinine (>84 mmol/l)**				
Yes	40 (22)	52 (26.1)	0.789	0.374
No	128 (70.3)	134 (67.3)		
**C-Reactive protein (>5 mmol/l)**				
Yes	134 (73.6)	153 (76.9)	0.021	0.886
No	22 (12.1)	24 (12.1)		
**Tumor resection of lung cancer**				
Yes	18 (10.4)	21 (10.6)	0.045	0.831
No	164 (90.1)	178 (89.4)		
**Chemotherapy**				
Yes	49 (81.9)	160 (80.4)	0.230	0.632
No	32 (18.5)	39 (19.6)		
**Platinum based chemotherapy**				
Yes	142 (78)	151 (75.9)	0.246	0.620
No	40 (22)	48 (24.1)		
**Radiation**				
Yes	55 (27.6)	54 (29.7)	0.198	0.656
No	142 (71.4)	126 (69.2)		

### Identification of potential risk factors for VTE by Univariate analysis

There is a statistically significant correlation between lymph node status (≥N2), presence of metastasis, active smoking, surgical history, hypertension, coronaropathy, and the occurrence of VTE.

The univariate comparison for the identification of potential risk factors for VTE is detailed in Table 2.

**Table 2.** Comparison of variables between patients without and with VTE in the development group.

Variables	Without VTE n=135	With VTE n=47	χ ^2^	p
**Sex**				
Female	25 (18.5)	5 (10.6)	1.573	0.210
Male	110 (83.5)	42 (89.4)		
**Age (≥65 years)**				
Yes	65 (48.1)	27 (57.4)	1.206	0.272
No	70 (51.9)	20 (42.6)		
**BMI (≥25 kg/m ^2^)**				
Yes	21 (15.6)	11 (23.4)	0.434	0.510
No	76 (56.3)	30 (63.8)		
**WHO status (≥2)**				
Yes	21 (15.6)	39 (83)	0.047	0.828
No	113 (83.7)	8 (17)		
**TNM stage**				
I-II	15 (11.1)	2 (4.3)	1.935	0.164
III-IV	120 (88.9)	45 (95.7)		
**Lymph node status (≥N2)**				
Yes	90 (66.7)	41 (87.2)		
No	45 (33.3)	6 (12.8)	7.312	**0.007**
**Metastasis**				
Yes	76 (56.3)	37 (78.8)	7.449	**0.006**
No	59 (43.7)	10 (21.3)		
**Histological type**				
Small-cell carcinoma	12 (8.9)	9 (19.1)	3.595	0.058
Poorly or undifferentiated	18 (13.3)	6 (12.8)	0.010	0.921
non-Small-cell carcinoma				
Squamous cell carcinoma	37 (27.4)	11 (23.4)	0.288	0.592
Adenocarcinoma	64 (47.4)	20 (42.6)	0.331	0.565
Large cell carcinoma	2 (1.5)	1 (2.1)	0.090	0.764
**Active smoking**				
Yes	69 (51.1)	35 (74.5)	9.236	**0.002**
No	66 (48.9)	12 (25.5)		
**Hypertension**				
Yes	34 (25.2)	46 (97.9)	11.933	**0.001**
No	101 (74.8)	1 (2.1)		
**Diabetes mellitus**				
Yes	21 (15.6)	5 (10.6)	0.688	0.407
No	114 (84.4)	42 (89.4)		
**Dyslipidemia**				
Yes	7 (5.2)	2 (95.7)	0.064	0.800
No	128 (94.8)	45 (4.3)		
**Coronaropathy**				
Yes	17 (12.6)	0 (0)	0.6528	**0.011**
No	118 (87.4)	47 (100)		
**Cardiac failure**				
Yes	6 (4.4)	0 (0)	2.160	0.142
No	129 (95.6)	47 (100)		
**Renal failure**				
Yes	3 (2.2)	0 (0)	1.062	0.303
No	132 (97.8)	47 (100)		
**History of surgery**				
Yes	98 (72.6)	6 (12.8)	4.142	**0.042**
No	37 (27.4)	41 (87.2)		
**WBC (≥10 × 10 ^9^/l)**				
Yes	52 (38.5)	18 (38.3)	0.004	0.951
No	82 (60.7)	29 (61.7)		
**Hb (<10 g/l)**				
Yes	14 (10.4)	8 (17)	1.451	0.228
No	121 (89.6)	39 (83)		
**Platelets (≥300 × 10 ^9^/l)**				
Yes	71 (52.6)	24 (51.1)	0.033	0.857
No	64 (47.4)	23 (48.9)		
**Creatinine (>84 mmol/l)**				
Yes	31 (23)	9 (19.1)	0.492	0.483
No	92 (68.1)	36 (76.6)		
**C-Reactive protein (>5 mmol/l)**				
Yes	94 (14.1)	40 (85.1)	2.488	0.115
No	19 (69.6)	3 (6.4)		
**Tumor resection of lung cancer**				
Yes	17 (12.6)	21 (10.6)	2.777	0.249
No	118 (87.4)	178 (89.4)		
**Chemotherapy**				
Yes	113 (83.7)	36 (23.4)	2.017	0.156
No	22 (16.3)	11 (76.6)		
**Platinum-based chemotherapy**				
Yes	110 (81.5)	32 (31.9)	3.649	0.056
No	25 (18.5)	15 (68.1)		
**Radiation**				
Yes	36 (26.7)	18 (38.3)	2.362	0.307
No	99 (73.3)	29 (61.7)		

### Development of the predictive risk score system for VTE by multivariate analysis

*Determination of risk score system items*

Variables with a significance level of p<0.250 were entered into the binary logistic regression.

A hemoglobin level <10 g/l, the presence of metastases, the histological type of poorly or undifferentiated NSCLC, and active smoking are the significant variables (p<0.05; therefore, they have been selected as the items of the score.

Based on the weight of the different regression coefficients, we established a risk score system as follows ( Table 3):

**Table 3.** Predictive factors for VTE determined from the development group by Multivariate analysis.

Variables	Score	B	Wald	p	OR, IC 95%
Hemoglobin level <10 g/l	+1	1.508	4.258	0.039	4.520 [1.079-18.937]
Presence of metastasis	+1	1.197	5.670	0.017	3.311 [1.236-8.871]
Histological type poorly or undifferentiated NSCLC	+2	1.875	6.390	0.011	6.522 [1.524-27.911]
Active smoking	+1	1.431	9.309	0.002	4.183 [1.668-10.488]

Total score=(hemoglobin level <10g/l: yes=+1/no=0) + presence of metastasis (yes=+1/no=0) + (histological type of poorly or undifferentiated NSCLC: yes=+2/no=0) + (active smoking: yes=+1/no=0).

The risk of VTE was significantly correlated with the risk score in the development group (Pearson contingency coefficient=26.757, p<10 ^-3^).

*Determination of risk groups*

The cut-off of our VTE predictive score was identified via the ROC curve. Indeed, a total score <2 allows the classification of patients in the “low risk of VTE” group. The other risk classes were developed on the basis of the predicted incidence of VTE.

As a result, patients were classified into 3 risk groups ( Table 4): “low risk” (score 0-1 [predicted incidence <29%, n=92]), “moderate risk” (score 2-3 [predicted incidence 29-43%, n=85]), and “high risk” (score 4-5 [predicted incidence >43%, n=5]).

**Table 4.** Classification of patients according to the predicted risk of the risk score and the actual incidence of VTE.

Score	Development group					Validation group				
	Predicted incidence	Number of patients	Actual incidence (%)	Risk group	PPV/NPV (%)	Predicted incidence	Number of patients	Actual incidence (%)	Risk group	PPV/NPV (%)
**0**	4.7%	25	2 (8%)	low risk (<29%, n=92)	NA/87 Se=NA Sp=100%	10.1%	30	4 (13.33%)	low risk (<29%, n=101)	NA/82 Se=NA Sp=100%
**1**	18.1%	67	10 (14.9%)			21.4%	71	14 (19.71%)		
**2**	38.8%	64	22 (34.4%)	Moderate risk (29-43%, n=85)	48/82.9 Se=80% Sp=52.7%	37.33%	76	27 (35.5%)	Moderate risk (29-43%, n=86)	80/63.3 Se=12.1% Sp=98%
**3**	53.1%	21	8 (38.1%)			40%	10	6 (60%)		
**4**	83.9%	5	5 (100%)	High risk (>43%, n=5)	100/NA Se=100% Sp=NA	35.47%	11	4 (36.3%)	High risk (>43%, n=12)	NA/66.7 Se=NA Sp=100
**5**	0 %	0	0 (0%)			48.7%	1	0 (0%)		

Abbreviations: NA: not applicable, PPV: positive predictive value, NPV: negative predictive value, Se: sensitivity, Sp: specificity.

**Figure 3.**
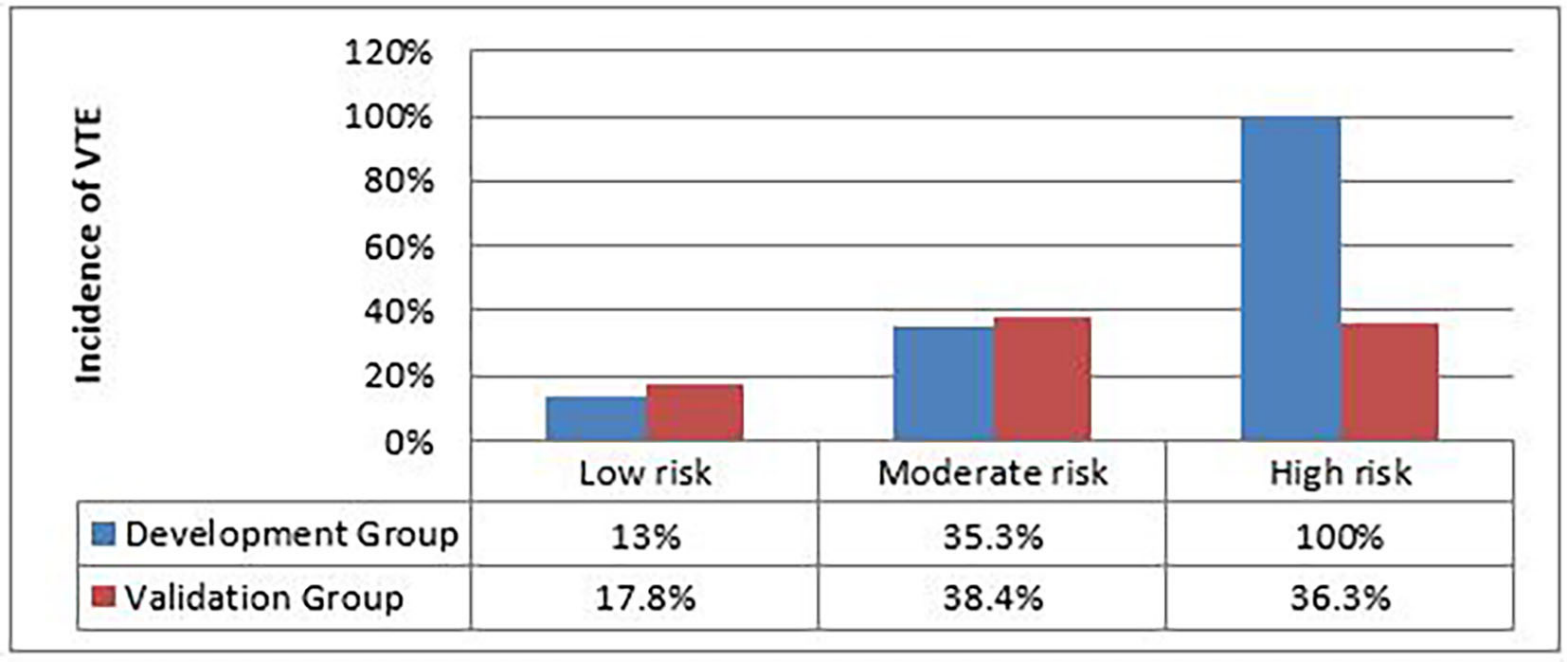
Distribution of VTE according to the 3 risk classes between the development group and the validation group.

The incidence of VTE according to the 3 risk classes in the development group and in the validation group is detailed in Figure 3.

The percentages of VTE in “low-risk” and “moderate-risk” patients are similar between the development and validation groups (low-risk: 13% vs 17.8%, moderate-risk: 35.3% vs 38.4%; respectively). In contrast, the percentage of VTE in “high-risk” patients is much higher in the development group compared to the patients in the validation group (100% vs 36.3%, p=0.012)

### Validation of VTE predictive score

*Internal validation*

**Figure 4.**
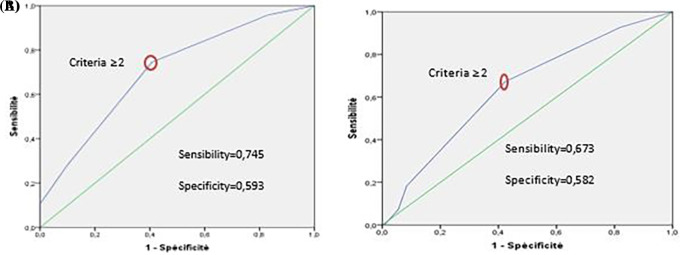
(A) ROC curve of VTE prediction model using the development group. (B) ROC curve of VTE prediction model using the validation group and after applying the bootstrap method (1000 samples).

In the development group, the risk score system has good discrimination. It can distinguish “high-risk” from “low-risk” VTE patients (c statistic=0.703 [0.618-0.789], ( Figure 4 A)). This prediction model also showed good calibration according to the Hosmer-Lemeshow test (χ ^2^=2.381, p=0.882).

*External validation*

The risk score system shows low discrimination in the validation group (c-statistics=0.641 [0.557-0.726], ( Figure 4)).

However, despite being poorly discriminating, it was well calibrated according to the Hosmer-Lemeshow test (χ ^2^= 6.250; p=0.396).

Considering the aforementioned classification, patients in the validation group were classified into 3 risk groups ( Table 4): “low risk” (score 0-1 [predicted incidence <29%, n=101), “moderate risk” (score 2-3 [predicted incidence 29-43%, n=86]), and “high risk” (score 4-5 [predicted incidence >43%, n=12).

## Discussion

We created a novel prediction model to assess the risk of venous thromboembolism (VTE) in patients diagnosed with lung cancer in the development cohort. Subsequently, we conducted an external validation of the model using a separate validation cohort. Our study involved a total of 381 patients, and our findings indicated that the prevalence of VTE in lung cancer patients was 26.8%, surpassing the rates reported in previous studies. ^9^^–^^11^

The variability in the incidence of VTE is due to several risk factors. In our study, lymph node status (≥N2), presence of metastasis, active smoking, surgical history, hypertension, and absence of coronaropathy were correlated with the occurrence of VTE. However, in other Tunisian studies, TNM stage IV and non-small squamous carcinoma were associated with high VTE incidence. ^9^^,^^10^

Thus, to reduce the occurrence of VTE, it is imperative to identify and assess all possible risk factors while determining the appropriate prophylactic measures for these patients. Various predictive models have been suggested to anticipate VTE occurrence in individuals with lung cancer. These models have incorporated several factors based on existing literature, and it is important to consider biomarkers associated with thrombosis. ^12^

The Khorana risk score, a widely recognized predictive scoring system, categorizes cancer patients into distinct risk groups and identifies a high-risk group for thromboprophylaxis. It is considered the prevailing and valuable tool for predicting VTE in the cancer population. ^12^^,^^13^

The majority of factors included in various risk scoring systems have been incorporated into our risk score system. However, we did not include the D-dimer test, despite its significance, due to the limited number of observed values in our dataset. ^13^ and, most importantly, due to the unavailability of this test in our current practice.

Furthermore, these aforementioned predictive risk scores were developed using data from patients diagnosed with various types of cancer, whereas our scoring system is specifically tailored to lung cancer patients. ^13^ Thus, lung cancer data were included in our score but not adopted in most models. Some of these variables were present in our definitive risk score (i.e., the presence of metastasis and histological type of poorly or undifferentiated NSCLC).

In previous retrospective studies reported in the literature, adenocarcinoma has been identified as a strong predictor of VTE onset. ^13^ Blom *et al.* conducted a study involving 537 NSCLC patients to investigate thrombotic risk and observed a 20-fold higher risk of VTE compared to the general population. Among the patients, those with adenocarcinoma had a three-fold higher risk (incidence=66.7%) than those with squamous cell carcinoma of the lung (incidence=21.2%). ^14^ Similarly, in another cohort of 493 NSCLC patients, Tagalakis *et al.* reported a high incidence of DVT (13.6%). ^15^ However, in our study, we found that poorly or undifferentiated NSCLC was associated with a higher prevalence of VTE, while adenocarcinoma was not predictive of the occurrence of VTE. ^13^ Blom *et al.* ^14^^,^^15^

Body mass index (BMI), which is incorporated in both the Khorana score and Caprini VTE risk assessment, was integrated into our risk system. However, we used a lower cut-off (≥25 kg/m ^2^) possibly due to the prevalence of poor nutritional status among lung cancer patients. ^5^^,^^16^

The hemogram parameter (hemoglobin, platelets, and leukocytes) included in the other risk score models was used in our study. Patients with a hemoglobin level <10 g/l had a four-fold higher risk of VTE. Other biomarkers, namely CRP and creatinine, were also included in our system; these biomarkers were not used by most VTE risk models. A cohort study investigating VTE risk among 3159 patients with newly diagnosed solid tumors concluded that elevated CRP and creatinine levels were predictive of VTE. ^17^

Our scoring system incorporates cancer therapy and surgery as variables. Previous studies have demonstrated that cancer therapy, including chemotherapy, antiangiogenic therapy, and hormonal therapy, increases the risk of VTE. ^18^^–^^21^ Christensen *et al.*, after reviewing 19 studies involving 10,660 patients with primary lung cancer undergoing curative-intent operations, found that the risk of VTE appears to be highest during the early postoperative period, with a subsequent decrease in risk. ^22^

As mentioned earlier, since risk factors vary from one population to another, there is a need to develop a risk score system specific to our population, in order to assist healthcare practitioners in developing appropriate prophylactic strategies for patients at risk of developing VTE.

After conducting logistic regression analysis in our study, we identified four items that were included in our risk score system. To the best of our knowledge, this risk score represents the first attempt to predict the potential incidence of VTE specifically for Tunisian patients with lung cancer. The developed risk score suggests that the incidence of VTE is expected to increase exponentially.

Scores below 2 were associated with a low risk of VTE, while scores of 4 or higher were associated with a high risk. The discriminant validity of this VTE score system was confirmed in the validation group, although there was a moderate decrease in predictive power. However, the classification of patients based on the score remained clinically meaningful. The notable variation in prognosis among the three risk groups should aid physicians in determining the appropriate therapeutic approach. Therefore, we strongly recommend thromboprophylaxis for Tunisian patients with moderate and high VTE risks.

Despite its poor predictive discrimination, this score presented several strengths. Firstly, to our knowledge, it is the first risk prediction model that included the occurrence of SVT as a predictable event. In fact, our decision to add SVT among outcomes was not arbitrary but based on several studies. A recent study conducted in 2022 highlighted the significance of SVT as a condition and revealed that patients with cancer and SVT are at an increased risk of thromboembolic complications. ^23^ In addition, Galanaud *et al.* suggested that cancer patients with SVT exhibit a poor prognosis, comparable to those with cancer-related DVT, with a heightened risk of recurrence of DVT-PE.

Secondly, we believe that this score can be applied to other populations whose characteristics are quite similar to those of our Tunisian population and with poor means on board, especially in underdeveloped countries. Nevertheless, it is essential to perform external validation of this score using data from diverse populations in order to ensure its generalizability and reliability.

The present study has several limitations that should be acknowledged. First, this study was based on 381 patients from three tertiary centers in northern Tunisia. Given the limited sample size, it is important to note that our patients may not adequately represent the diversity of our population. Secondly, we did not conduct an assessment of the reproducibility of our risk score in the prospective validation cohort. Thirdly, it is important to consider that personal and family history of VTE, as well as the use of anticoagulant or antiplatelet treatment at the time of lung cancer diagnosis, could potentially impact our findings. Finally, it should be noted that the cut-off values for the potential VTE variables included in our risk model were determined based on clinical experience or existing literature, rather than individualized threshold values determined by ROC curve analysis.

We have tried to respond to a need specific to the characteristics of our country where the economic crisis makes it very difficult to provide care according to international standards. Our score contains simple items available to any Tunisian practitioner.

The collection of the four items is straightforward: smoking history can be obtained through an interview, a complete blood count (CBC) is a readily available test in Tunisia, even in primary care settings, determining the histological type, and conducting staging assessments are commonly practiced.

## Conclusion

In conclusion, the frequency of VTE in this study was high, at 26.8%. A predictive score for VTE was developed and validated by including epidemiological, clinical, and biological data. This score, despite its low discrimination, has a good positive and negative predictive value for a moderate risk of VTE. The stratification of risk in this newly developed risk system may guide the clinician in prescribing preventive treatment for VTE. However, our study has limitations, particularly the retrospective nature of the analysis and the small sample size. Hence, the need to conduct a prospective study on a nationwide scale for the validation of this score.

## Data Availability

Figshare: Rouis, Houda (2023). Development and Validation of a Risk Score System for Early Prediction of Venous Thromboembolism in Patients with Lung Cancer. figshare. Dataset. https://doi.org/10.6084/m9.figshare.23582829.v1. ^24^ Data are available under the terms of the Creative Commons Attribution 4.0 International license (CC-BY 4.0).

## References

[1] Khorana AA Palaia J Rosenblatt L : Venous thromboembolism incidence and risk factors associated with immune checkpoint inhibitors among patients with advanced non-small cell lung cancer. *J Immunother Cancer.* 1 janv 2023;11(1):e006072. 10.1136/jitc-2022-006072 36657815 PMC9853260

[2] Bagchi A Khan MS Saraswat A : Increased Incidence of Thrombotic Complications With Non-small Cell Lung Cancer Necessitates Consideration of Prophylactic Anticoagulation in Young Individuals. *Cureus.* sept 2021;13(9):e17769. 10.7759/cureus.17769 34659980 PMC8494503

[3] Khorana AA Kuderer NM McCrae K : Cancer associated thrombosis and mortality in patients with cancer stratified by khorana score risk levels. *Cancer Med.* nov 2020;9(21):8062–8073. 10.1002/cam4.3437 32954653 PMC7643641

[4] Timp JF Braekkan SK Versteeg HH : Epidemiology of cancer-associated venous thrombosis. *Blood.* 5 sept 2013;122(10):1712–1723. 10.1182/blood-2013-04-460121 23908465

[5] Khorana AA : Venous thromboembolism and prognosis in cancer. *Thromb. Res.* juin 2010;125(6):490–493. 10.1016/j.thromres.2009.12.023 20097409 PMC2878879

[6] Galanaud JP Blaise S Sevestre MA : Long-term outcomes of isolated superficial vein thrombosis in patients with active cancer. *Thromb. Res.* nov 2018;171:179–186. 10.1016/j.thromres.2018.04.013 29789147

[7] Comparison of risk prediction scores for venous thromboembolism in cancer patients: a prospective cohort study - PMC. [cité 5 juin 2023]. Reference Source 10.3324/haematol.2017.169060 PMC5685240 28550192

[8] Pourhoseingholi MA Vahedi M Rahimzadeh M : Sample size calculation in medical studies. PMC4017493 24834239

[9] Racil H Laaribi G Cherif H : Lung cancer with venous thrombo-embolism: clinical characteristics. *Tunis. Med.* juill 2011;89(7):616–620. 21780036

[10] Ketata W Moussa N Bahloul N : MALADIE VEINEUSE THROMBOEMBOLIQUE ET CANCER BRONCHIQUE: A PROPOS D’UNE SERIE TUNISIENNE VENOUS THROMBOEMBOLISM AND LUNG CANCER: ABOUT A TUNISIAN SERIES.

[11] Chew HK Davies AM Wun T : The incidence of venous thromboembolism among patients with primary lung cancer. *J Thromb Haemost.* 1 avr 2008;6(4):601–608. 10.1111/j.1538-7836.2008.02908.x 18208538

[12] Di W Xu H Xue T : Advances in the Prediction and Risk Assessment of Lung Cancer-Associated Venous Thromboembolism. *Cancer Manag Res.* 31 déc 2021;13:8317–8327. 10.2147/CMAR.S328918 34764694 PMC8575248

[13] Li Z Zhang G Zhang M : Development and Validation of a Risk Score for Prediction of Venous Thromboembolism in Patients With Lung Cancer. *Clin Appl Thromb Hemost.* 12 mars 2020;26:1076029620910793. 32162530 10.1177/1076029620910793 PMC7288811

[14] Blom JW Osanto S Rosendaal FR : The risk of a venous thrombotic event in lung cancer patients: higher risk for adenocarcinoma than squamous cell carcinoma. *J Thromb Haemost.* oct 2004;2(10):1760–1765. 10.1111/j.1538-7836.2004.00928.x 15456487

[15] Tagalakis V Levi D Agulnik JS : High risk of deep vein thrombosis in patients with non-small cell lung cancer: a cohort study of 493 patients. *J Thorac Oncol.* août 2007;2(8):729–734. 10.1097/JTO.0b013e31811ea275 17762339

[16] Caprini JA : Risk assessment as a guide for the prevention of the many faces of venous thromboembolism. *Am J Surg.* janv 2010;199(1 Suppl):S3–S10. 10.1016/j.amjsurg.2009.10.006 20103082

[17] Haltout J Awada A Paesmans M : Predictive factors for cancer-associated thrombosis in a large retrospective single-center study. *Support Care Cancer.* avr 2019;27(4):1163–1170. 10.1007/s00520-018-4602-6 30610431

[18] Ando Y Hayashi T Sugimoto R : Risk factors for cancer-associated thrombosis in patients undergoing treatment with immune checkpoint inhibitors. *Investig New Drugs.* août 2020;38(4):1200–1206. 10.1007/s10637-019-00881-6 31823160 PMC7340643

[19] Khorana AA Francis CW Culakova E : Risk factors for chemotherapy-associated venous thromboembolism in a prospective observational study. *Cancer.* 15 déc 2005;104(12):2822–2829. 10.1002/cncr.21496 16284987

[20] Nalluri SR Chu D Keresztes R : Risk of venous thromboembolism with the angiogenesis inhibitor bevacizumab in cancer patients: a meta-analysis. *JAMA.* 19 nov 2008;300(19):2277–2285. 10.1001/jama.2008.656 19017914

[21] Behrendt CE Ruiz RB : Venous thromboembolism among patients with advanced lung cancer randomized to prinomastat or placebo, plus chemotherapy. *Thromb Haemost.* oct 2003;90(4):734–737. 14515196 10.1160/TH03-01-0041

[22] Christensen TD Vad H Pedersen S : Venous thromboembolism in patients undergoing operations for lung cancer: a systematic review. *Ann Thorac Surg.* févr 2014;97(2):394–400. 10.1016/j.athoracsur.2013.10.074 24365217

[23] Hirmerová J Seidlerová J Šubrt I : Prevalence of cancer in patients with superficial vein thrombosis and its clinical importance. *J Vasc Surg Venous Lymphat. Disord.* janv 2022;10(1):26–32. 10.1016/j.jvsv.2021.05.006 34089942

[24] Rouis H : Development and Validation of a Risk Score System for Early Prediction of Venous Thromboembolism in Patients with Lung Cancer.Dataset. *figshare.* 2023. 10.6084/m9.figshare.23582829.v1

