# Supplementary material for: A novel risk score for venous thromboembolism in lung cancer patients: a retrospective cohort study
Source: F1000Res. 2025 Aug 18;12:1388. Originally published 2023 Oct 20. [Version 2] doi: 10.12688/f1000research.138878.2 (PMC12910196; doi:10.12688/f1000research.138878.2)
Supplement: Peer review history

# Reviewer report: Reviewer response for version 2

**DOI:** 10.5256/f1000research.186248.r455484

**Published:** 2026-02-16

**Authors:** Marc Vasse

**Affiliations:** 1 Université Paris-Saclay, Foch Hospital, Suresnes, France

In this paper, the authors describe a new score to identify the thrombotic risk in lung cancer patients treated by "classical chemotherapy" used in low-to-middle-income countries.

 1) The introduction is too long and bibliography is not relevant. The frequency of thrombosis in lung cancer patients is not so high (20 to 30 %) as indicated, and the reference 1 applies to patients treated by check point inhibitors, that is not the case of the patients of this study.

 2) I am surprised that patients are divided (fig 1) as only PE. No patients had both deep venous thrombosis and pulmonary embolism?

 3) All cases of VTE are indicated as 102. Surprisingly, the sum of "only PE" + "only DVT " + only SVT is 108.

 4) The authors indicate in the discussion that their score as a poor predictive value. Usually, despite a poor discriminating capacity in lung cancer patients, the Khorana score (KS) is used. A comparison of the Khorana score with this new score could be interesting. In addition, in lung cancer, KS is predictive of mortality (PMID 29806470). Is your score predictive of the mortality?

Is the work clearly and accurately presented and does it cite the current literature?

Partly

If applicable, is the statistical analysis and its interpretation appropriate?

I cannot comment. A qualified statistician is required.

Are all the source data underlying the results available to ensure full reproducibility?

Yes

Is the study design appropriate and is the work technically sound?

Partly

Are the conclusions drawn adequately supported by the results?

Partly

Are sufficient details of methods and analysis provided to allow replication by others?

Yes

Reviewer Expertise:

Coagulation, cancer associated thrombosis

I confirm that I have read this submission and believe that I have an appropriate level of expertise to confirm that it is of an acceptable scientific standard, however I have significant reservations, as outlined above.

# Reviewer report: Reviewer response for version 2

**DOI:** 10.5256/f1000research.186248.r406432

**Published:** 2025-08-23

**Authors:** Georgia Gomatou

**Affiliations:** 1 Oncology Unit, Third Department of Medicine, “Sotiria” General Hospital for Diseases of the Chest, National and Kapodistrian University of Athens, Athens, Greece; 2 National and Kapodistrian University of Athens School of Medicine, Athens, Attica, Greece

The authors have successfully incorporated the reviewers' suggestions. No further comments.

Is the work clearly and accurately presented and does it cite the current literature?

Yes

If applicable, is the statistical analysis and its interpretation appropriate?

Yes

Are all the source data underlying the results available to ensure full reproducibility?

Yes

Is the study design appropriate and is the work technically sound?

Yes

Are the conclusions drawn adequately supported by the results?

Yes

Are sufficient details of methods and analysis provided to allow replication by others?

Yes

Reviewer Expertise:

Medical Oncology, Thoracic Oncology

I confirm that I have read this submission and believe that I have an appropriate level of expertise to confirm that it is of an acceptable scientific standard.

# Reviewer report: Reviewer response for version 1

**DOI:** 10.5256/f1000research.152109.r335912

**Published:** 2024-11-06

**Authors:** Georgia Gomatou

**Affiliations:** 1 Oncology Unit, Third Department of Medicine, “Sotiria” General Hospital for Diseases of the Chest, National and Kapodistrian University of Athens, Athens, Greece; 2 National and Kapodistrian University of Athens School of Medicine, Athens, Attica, Greece

This is an original study describing a prediction score of VTE in patients with lung cancer. In general, the manuscript is well-written and the methods and results are clearly presented. My suggestions in order to improve the paper:

 1. Why are the non-inclusion criteria described seperately from exclusion criteria? Please combine.

 2. The limitations should be included in the Discussion section (last paragraph of Discussion) and not in the Conclusions. 

 3. In Figure 1 you use the term 'randomization'. However, the process of randomly allocating patients in each group is not randomization. Please delete the term.

Is the work clearly and accurately presented and does it cite the current literature?

Yes

If applicable, is the statistical analysis and its interpretation appropriate?

Yes

Are all the source data underlying the results available to ensure full reproducibility?

Yes

Is the study design appropriate and is the work technically sound?

Yes

Are the conclusions drawn adequately supported by the results?

Yes

Are sufficient details of methods and analysis provided to allow replication by others?

Yes

Reviewer Expertise:

Medical Oncology, Thoracic Oncology

I confirm that I have read this submission and believe that I have an appropriate level of expertise to confirm that it is of an acceptable scientific standard, however I have significant reservations, as outlined above.

# Response

**Published:** 2025-08-06

**Authors:** CHIRINE MOUSSA

**Affiliations:** Abderrahmen Mami Pneumology and Phthisiology Hospital, Ariana, Ariana, Tunisia

**1. Why are the non-inclusion criteria described seperately from exclusion criteria? Please combine.**

**Response to the reviewer:**

 Thank you for your valuable comment regarding the separation of non-inclusion and exclusion criteria.

 In our protocol, we initially presented them separately to reflect the different stages of patient selection: non-inclusion criteria correspond to conditions preventing initial eligibility (e.g., lack of histological confirmation), while exclusion criteria relate to factors identified after preliminary assessment (e.g., comorbidities, acute events, or unusable records).

 However, to improve clarity and according to your suggestion, we are happy to combine both into a single section titled “Non-inclusion and exclusion criteria” for simplicity and better readability.

 Proposed combined criteria:

- Histologically unconfirmed bronchopulmonary carcinoma (BPC)
- Secondary pulmonary localizations
- History of:
   • Other neoplasms
   • Acute phase of myocardial infarction (MI)
   • Acute phase of stroke
- Unusable medical records

**2. The limitations should be included in the Discussion section (last paragraph of Discussion) and not in the Conclusions.**

**Response to reviewer:**

 Thank you for your valuable suggestion. We agree that discussing the study’s limitations is more appropriate in the Discussion section. We confirm that these limitations are already addressed within a dedicated paragraph of the Discussion. Accordingly, we will remove the sentence referring to the limitations from the Conclusions section to avoid redundancy and improve the overall clarity of the manuscript.

**3. In Figure 1 you use the term 'randomization'. However, the process of randomly allocating patients in each group is not randomization. Please delete the term.**

**Response to the reviewer:**

 Thank you for your valuable comment regarding the use of the term “randomization” in Figure 1.

 We acknowledge that the allocation process used in our study was based on generating “random” numbers from the sequence of patients’ medical record numbers. This method does not constitute a strict randomization procedure as per clinical trial standards, since medical record numbers are assigned sequentially and may introduce allocation bias.

 Therefore, to avoid any misunderstanding, we have removed the term “randomization” from Figure 1 and replaced it with “allocation” or a similar neutral term that better reflects the actual process.

 We appreciate your careful review and hope this clarifies the method used.

# Reviewer report: Reviewer response for version 1

**DOI:** 10.5256/f1000research.152109.r253369

**Published:** 2024-05-15

**Authors:** Ping Wang

**Affiliations:** 1 The Fourth Hospital of Hebei Medical University, Shijiazhuang, China

Many literatures have reported that the incidence rate of tumor with VTE is significantly higher than that of other diseases, and the incidence rate of lung cancer with VTE is higher. There are many studies on the risk factors of lung cancer combined with VTE, and many suggest that pathological types, stages, and other factors are related to the occurrence of VTE. This manuscript introduces several processes for establishing a VTE screening model, but some issues still need to be discussed.

 1. The enrolled patients have a large annual span of nearly ten years. The treatment of lung cancer has also undergone significant changes over the past decade. When were patients enrolled in each group? How to maintain consistency in the inclusion criteria for patients over the past decade, especially whether current immune checkpoint inhibitors and anti angiogenic drugs have an impact on VTE?

 2. What is the difference between chemotherapy and platinum based chemotherapy? Does chemotherapy include chemotherapy with platinum containing drugs?

 3. You collect relevant data and blood tests for enrolled patients. At what time point were the BMI, WBC, Hb, Platelets, Creatinine, and CRP data collected from patients? Especially for non VTE patients, which time point data is appropriate?

 4. Is there any basis researches for the predicted score in Table 3? The P-value for active smoking is the lowest, why is it only 1 point, while the Historical type poor or unidentified NSCLC is 2 points?

 5. In univariate analysis the result of hemoglobin level<10 g/l was P=0.228, while in multivariate analysis was P=0.039? What are the criteria for incorporating multiple factor analysis and comparison?

Is the work clearly and accurately presented and does it cite the current literature?

Partly

If applicable, is the statistical analysis and its interpretation appropriate?

I cannot comment. A qualified statistician is required.

Are all the source data underlying the results available to ensure full reproducibility?

Partly

Is the study design appropriate and is the work technically sound?

Partly

Are the conclusions drawn adequately supported by the results?

Partly

Are sufficient details of methods and analysis provided to allow replication by others?

Partly

Reviewer Expertise:

Lung cancer and VTE, lung cancer and infection.

I confirm that I have read this submission and believe that I have an appropriate level of expertise to confirm that it is of an acceptable scientific standard, however I have significant reservations, as outlined above.

# Response

**Published:** 2025-08-06

**Authors:** CHIRINE MOUSSA

**Affiliations:** Abderrahmen Mami Pneumology and Phthisiology Hospital, Ariana, Ariana, Tunisia

**1. The enrolled patients have a large annual span of nearly ten years. The treatment of lung cancer has also undergone significant changes over the past decade. When were patients enrolled in each group? How to maintain consistency in the inclusion criteria for patients over the past decade, especially whether current immune checkpoint inhibitors and anti angiogenic drugs have an impact on VTE?**

**Response to reviewer**

 Thank you for this relevant comment. We acknowledge that lung cancer management has significantly evolved over the past decade, particularly with the introduction of immune checkpoint inhibitors and targeted therapies. However, it is important to note that in Tunisia, a low-to-middle-income country, access to these innovative therapies remains extremely limited. Immunotherapy and targeted treatments have only been introduced into clinical practice in the past two years, and only a restricted number of molecules are currently available, due to their high cost and limited accessibility within the public health system.

 Consequently, throughout the study period, which spans nearly ten years, the vast majority of patients included were treated with conventional chemotherapy protocols. This contributes to the overall consistency of treatment regimens across the cohort. Moreover, at the time of inclusion, no patients in the VTE group had received immune checkpoint inhibitors or anti-angiogenic agents before the thromboembolic event.

 Thus, the potential impact of these newer therapies on the incidence of VTE was not a confounding factor in our analysis. We have now clarified this point in the revised manuscript.

**2. What is the difference between chemotherapy and platinum based chemotherapy? Does chemotherapy include chemotherapy with platinum containing drugs?**

**Response to reviewer:**

 Thank you for your insightful question. In our manuscript, we made a specific distinction between *chemotherapy* in general and *platinum-based chemotherapy* because of the well-established association between platinum compounds and increased risk of venous thromboembolism (VTE), particularly in lung cancer. This is why platinum-based chemotherapy is explicitly included in risk assessment tools such as the PROTECHT score.

 To clarify:

- **“Chemotherapy”** is a broad term that refers to any cytotoxic anticancer treatment, regardless of the drug class.
- **“Platinum-based chemotherapy”** refers specifically to regimens that include platinum compounds, such as cisplatin or carboplatin.

Therefore, while platinum-based chemotherapy is indeed a subset of chemotherapy, we chose to identify it separately in our analysis due to its particular thrombotic profile and relevance to the pathophysiology of VTE in lung cancer patients.

**3. You collect relevant data and blood tests for enrolled patients. At what time point were the BMI, WBC, Hb, Platelets, Creatinine, and CRP data collected from patients? Especially for non VTE patients, which time point data is appropriate?**

**Response to reviewer:**

 Thank you for your question. For all enrolled patients, including those without VTE, the data on BMI, white blood cell count (WBC), hemoglobin (Hb), platelet count, creatinine, and C-reactive protein (CRP) were collected at the time of the first hospital admission, immediately after the confirmation of lung cancer diagnosis and prior to the initiation of any treatment, regardless of treatment type. This approach ensured uniformity in data collection and minimized the influence of treatment-related changes on baseline biological parameters.

 We have now clarified this point in the revised version of the manuscript.

**4. Is there any basis researches for the predicted score in Table 3? The P-value for active smoking is the lowest, why is it only 1 point, while the Historical type poor or unidentified NSCLC is 2 points?**

**Response to reviewer:**

 Thank you for your valuable comment.

 The point allocation in Table 3 was not based solely on the statistical significance (p-values), but rather on the β coefficients obtained from multivariate logistic regression, which reflect the strength of association between each variable and the outcome (VTE occurrence). To construct the score, we applied a commonly used method in predictive model development, which involves:

1. Dividing each β coefficient by the smallest absolute β coefficient in the model (used as a reference),
2. Rounding the result to the nearest integer to assign the corresponding weight (score point).

In our model, the smallest β coefficient was that of “Presence of metastases”, with a value of 1.197. We then applied the following calculations:

- Hemoglobin < 10 g/dL: 1.508 / 1.197 = 1.26, rounded to 1 point
- Presence of metastases: 1.197 / 1.197 = 1, rounded to 1 point
- Histological type difficult to classify (NSCLC): 1.875 / 1.197 = 1.57, rounded to 2 points
- Active smoking: 1.431 / 1.197 = 1.19, rounded to 1 point

Therefore, even though *active smoking* had a low p-value, its β coefficient was not the highest among the predictors. The histological type variable had the largest coefficient and thus received the highest weight (2 points). This approach ensures that the scoring reflects the relative contribution of each predictor rather than just their individual statistical significance.

**5. In univariate analysis the result of hemoglobin level<10 g/l was P=0.228, while in multivariate analysis was P=0.039? What are the criteria for incorporating multiple factor analysis and comparison?**

**Response to reviewer:**

 Thank you for raising this important point.

 It is not uncommon for a variable to be non-significant in univariate analysis but become statistically significant in multivariate analysis. In our case, the hemoglobin level <10 g/dL had a p-value of **0**.228 in univariate analysis, yet became statistically significant (p = 0.039) in multivariate analysis.

 This phenomenon may be explained by the presence of confounding or interaction effects between variables. In univariate analysis, each variable is assessed in isolation, without adjusting for the influence of other potential covariates. In contrast, multivariate analysis accounts for the combined and adjusted effects of multiple predictors. Once other variables are included in the model — such as metastatic status, histological subtype, and smoking — the independent contribution of hemoglobin to VTE risk becomes more evident.

 Moreover, the decision to retain variables for multivariate modeling was based not only on statistical thresholds but also on epidemiological and clinical relevance. In line with the approach proposed by Bursac et al. (2008), we included variables with univariate p-values below 0.250, as this cutoff has been shown to reduce the risk of omitting important variables too early. This strategy was also recommended by the epidemiologists of our institution during the study design phase.

 References:

  ( *Source:* *Bursac Z, Gauss CH, Williams DK, Hosmer DW. Purposeful selection of variables in logistic regression. Source Code Biol Med. 2008 Dec 16;3:17. doi: 10.1186/1751-0473-3-17. PMID: 19087314; PMCID: PMC2633005.*) The reference was included as the ninth entry in the reference list; however, the remaining references were not updated accordingly.

 Therefore, although the univariate association between low hemoglobin and VTE was not statistically significant, we retained it in the multivariate model due to its known pathophysiological plausibility in cancer-associated thrombosis, and its potential contribution to a more robust predictive model. After adjustment for other covariates, the association became statistically significant.

 We have added a clarification to this effect in the revised Methods section.
